# Effects of Atmospheric CO_2_ and Temperature on Wheat and Corn Susceptibility to *Fusarium graminearum* and Deoxynivalenol Contamination

**DOI:** 10.3390/plants10122582

**Published:** 2021-11-25

**Authors:** William T. Hay, Susan P. McCormick, Martha M. Vaughan

**Affiliations:** USDA, Agricultural Research Service, National Center for Agricultural Utilization Research, Mycotoxin Prevention and Applied Microbiology Research Unit, 1815 N. University Street, Peoria, IL 61604, USA; susan.mccormick@usda.gov (S.P.M.); martha.vaughan@usda.gov (M.M.V.)

**Keywords:** wheat, corn, *Fusarium graminearum*, climate change, elevated CO_2_, mycotoxins, deoxynivalenol

## Abstract

This work details the impact of atmospheric CO_2_ and temperature conditions on two strains of *Fusarium graminearum,* their disease damage, pathogen growth, mycotoxin accumulation, and production per unit fungal biomass in wheat and corn. An elevated atmospheric CO_2_ concentration, 1000 ppm CO_2_, significantly increased the accumulation of deoxynivalenol in infected plants. Furthermore, growth in cool growing conditions, 20 °C/18 °C, day and night, respectively, resulted in the highest amounts of pathogen biomass and toxin accumulation in both inoculated wheat and corn. Warm temperatures, 25 °C/23 °C, day and night, respectively, suppressed pathogen growth and toxin accumulation, with reductions as great as 99% in corn. In wheat, despite reduced pathogen biomass and toxin accumulation at warm temperatures, the fungal pathogen was more aggressive with greater disease damage and toxin production per unit biomass. Disease outcomes were also pathogen strain specific, with complex interactions between host, strain, and growth conditions. However, we found that atmospheric CO_2_ and temperature had essentially no significant interactions, except for greatly increased deoxynivalenol accumulation in corn at cool temperatures and elevated CO_2_. Plants were most susceptible to disease damage at warm and cold temperatures for wheat and corn, respectively. This work helps elucidate the complex interaction between the abiotic stresses and biotic susceptibility of wheat and corn to *Fusarium graminearum* infection to better understand the potential impact global climate change poses to future food security.

## 1. Introduction

*Fusarium graminearum* (*F. graminearum*) is a devastating mycotoxigenic fungal pathogen that can cause disease in cereal crops such as wheat and corn [1]. The pathogen is particularly destructive due to its production of trichothecene mycotoxins [2]. The trichothecene deoxynivalenol (DON) not only causes plant cell death but is also toxic to animals/humans and can cause vomiting, feed refusal, immunosuppression, and organ damage [3]. DON is a serious food safety concern because it remains stable in harvested grains, and is not destroyed during typical food processing, including cooking, baking, or brewing [4,5]. Thus, heavily contaminated grain must be removed from the food chain resulting in approximately 2 billion dollars in annual agroeconomic losses [6,7].

The severity of Fusarium epidemics and the accumulation of DON in cereal grains are strongly associated with weather, and climate change is predicted to increase the risk of disease in many grain growing regions of the world [8,9]. Infection typically occurs when conditions are warm and wet during flowering and seed fill [10]. However, the impact of weather conditions is also dependent on the *F. graminearum* isolate causing disease in the crop host.

The optimal temperature for DON production is on average approximately 25 °C, but can vary among different DON producing *Fusarium* species or *F. graminearum* isolates which are capable of producing DON at a wide range of temperatures from 15–30 °C [11,12]. In North America there are distinct populations of *F. graminearum* [13,14]. North American 1 (NA1) represents an endemic genetically diverse population that predominantly produces the trichothecene toxin analog 15-acetyldeoxynivalenol (15ADON). A more homogeneous invasive population, referred to as the North American 2 (NA2) population that produces 3-acetyldeoxynivalenol (3ADON), is thought to be displacing NA1 in certain regions [15]. While relatively few studies have compared the effects of abiotic variables on the different *F. graminearum* populations, in response to heat or cold treatments NA2 isolates exhibited increased DON production in comparison to NA1 isolates [16]. Additionally, *F. graminearum* isolates acclimated to elevated carbon dioxide (CO_2_) became more aggressive to wheat and caused more severe Fusarium head blight (FHB) and DON contamination in comparison to ambient CO_2_-acclimated isolates [16].

The effects of abiotic variable treatments on disease and mycotoxin accumulation are likely influenced by the medium/host. Changes in wheat nutritional content, due to growth at elevated [CO_2_], reduced *F. graminearum* growth but caused strain specific increases in mycotoxin production [17]. At elevated CO_2,_ *F. graminearum* radial growth was inhibited on artificial media [18], but conversely fungal biomass accumulation increased in infected wheat heads [19]. Additionally, the influence of elevated CO_2_ on *F. graminearum* aggressiveness, disease severity, and mycotoxin accumulation was shown to be dependent on both the interacting *F. graminearum* strain and wheat host variety [16,19,20]. Furthermore, it is well established that wheat and corn plants respond differently to temperature and elevated atmospheric CO_2_ because they have different photosynthetic systems. Warmer temperatures and elevated CO_2_ have a greater effect on C3 photosynthetic crops, such as wheat, than C4 photosynthetic crops, such as corn [21]. Unlike C3 crops which have lower photosynthetic efficiency at warmer temperatures or low intracellular concentrations of CO_2_ due to photorespiration [22], C4 plants have a unique anatomy (Kranz anatomy) which concentrates CO_2_ within the bundle sheath at the primary site of C4 photosynthesis, effectively eliminating photorespiration regardless of atmospheric temperature and CO_2_ concentration [23,24]. Thus, due to changes in photosynthetic efficiency, wheat plants typically experience changes in primary metabolism at warmer temperatures and elevated CO_2_, while corn does not. Thus, altering the pathogen host/growth medium.

Therefore, we hypothesized that it would be essential to include diverse interacting organisms and abiotic factors to fully understand the combined effects on the outcome of *F. graminearum* disease and mycotoxin contamination potential. To test this hypothesis, we compared disease development and DON contamination in a full-factorial experimental design using two *F. graminearum* strains (13MN1-6, 12SD6-2 representing an NA1 and NA2 strain, respectively: Table 1), two CO_2_ concentrations (400 ppm (ambient) and 1000 ppm (elevated)), and two temperature treatments (20 °C/18 °C (cool) and 25 °C/23 °C (warm)) temperature conditions.

Furthermore, we independently conducted these comparisons in two different hosts (wheat and corn). To accommodate the size of the experiment and utilize the same temperature treatments for both crops, we used two model varieties. Apogee is a full-dwarf hard red spring wheat (*Triticum aestivum*) cultivar developed in collaboration with the National Aeronautics and Space Administration (NASA) for growth in space, but has since also been established as a model wheat cultivar for FHB studies [27,28]. Gaspe Flint is a short season corn (*Zea mays*) variety originating from Canadian landrace which is adapted to cooler temperatures and has been used experimentally to evaluated Fusarium ear rot [29]. Characterizing the complex interaction between abiotic stress and biotic susceptibility to *F. graminearum* infection in wheat and corn will help elucidate the potential impact global climate change poses to future food security.

## 2. Results

**Figure 1:**
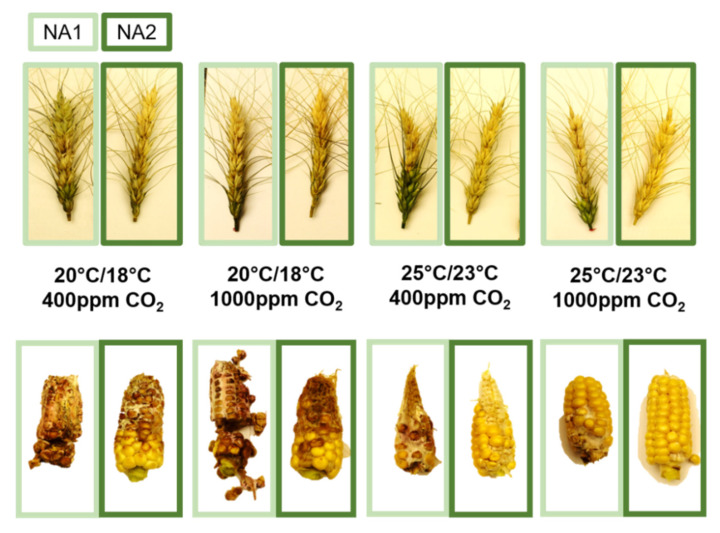
Representative *Fusarium graminearum* disease severity at elevated carbon dioxide and temperature on Apogee wheat and Gaspe Flint corn 21 and 17 days after inoculation, respectively. Wheat and corn inoculated with the NA1 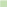 strain 13MN1-6, and the NA2 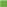 strain 12SD6-2.

The impact of elevated CO_2_ and temperature on disease severity was found to be host and pathogen dependent (Figure 1). Additionally, multiple factor interactions were found to influence disease, fungal biomass, mycotoxin accumulation, and toxin production per unit biomass.

### 2.1. Effects of Elevated CO_2_ and Temperature on F. graminearum Disease Severity in Wheat

**Figure 2:**
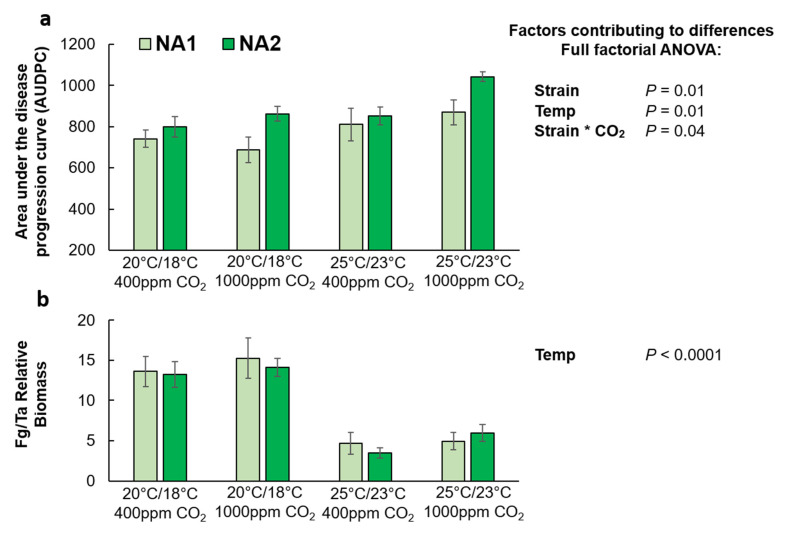
Disease progression (**a**) and relative fungal biomass (*Fg/Ta*) (**b**) in *Fusarium graminearum* inoculated Apogee wheat grown at ambient and elevated carbon dioxide and temperatures. Wheat inoculated with the NA1 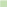 strain 13MN1-6, and the NA2 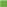 strain 12SD6-2 (*n* = 12). Interaction plots were generation in JMP demonstrating correlations (Supplemental Figure S1).

According to the 2 (temperature: 20 °C/18 °C and 25 °C/23 °C) × 2 (CO_2_:400 ppm and 1000 ppm) × 2 (strain: 13MN1-6 and 12SD6-2) full-factorial analysis of variance (ANOVA), significant contributing factors to differences in the area under the disease progression curve (AUDPC) in wheat included strain, temperature, and the interaction between strain and CO_2_. Visual disease progression was greater for the NA2 (12SD6-2) strain than the NA1 (13MN1-6) strain (*p* = 0.01; Figure 2a), and the warmer temperature treatment (25 °C/23 °C) resulted in significantly more visual disease symptoms (*p* = 0.01). Additionally, elevated CO_2_ significantly increased disease progression for the NA2 strain, but not the NA1 strain (*p* = 0.04). Interestingly, quantitative polymerase chain reaction (PCR) estimates of relative *F. graminearum* DNA to wheat DNA (designated as Fg/Ta relative biomass or Fg biomass) did not correspond with visual disease. Only temperature was a significant contributing factor, and *F. graminearum* biomass was 2 to 3.5-fold higher at the cool temperature treatment (20 °C/18 °C) in comparison to the warm treatment (*p* < 0.0001) (Figure 2b). Elevated CO_2_ did not affect *F. graminearum* biomass accumulation in wheat.

**Figure 3:**
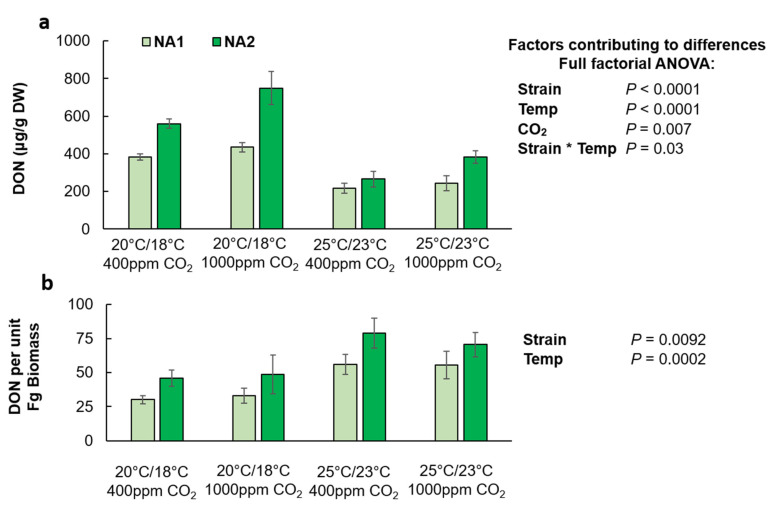
DON contamination level (**a**) and DON per unit fungal biomass (Fg) (**b**) in *Fusarium graminearum* inoculated Apogee wheat grown at ambient and elevated carbon dioxide and temperature. Wheat inoculated with the NA1 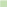 strain 13MN1-6, and the NA2 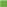 strain 12SD6-2 (*n* = 12). Interaction plots were generated in JMP demonstrating correlations (Supplemental Figure S2).

Differences in wheat DON contamination levels were significantly affected by strain, temperature, CO_2,_ and the interaction between strain and temperature (Figure 2a). Overall, NA2 inoculated wheat had more DON (*p* < 0.0001). However, this strain-specific difference was temperature dependent and although on average the NA2 strain resulted in more DON contamination, the difference was only significantly different at the cool temperature treatment (*p* = 0.03). The warm temperature treatment resulted in significantly less DON contamination (*p* < 0.0001), and elevated CO_2_ caused significantly greater DON contamination in inoculated wheat (27%; *p* = 0.007). Consistent with DON contamination levels, the amount of DON produced per unit fungal biomass for the NA2 strain was 40% more than the NA1 strain (Figure 3b; *p* = 0.0092). However, both strains had greater DON per unit biomass at the warm temperature treatment (*p* = 0.0002). There was no significant impact of elevated CO_2_ on DON production per unit biomass for either of the strains in infected wheat.

### 2.2. Effects of Elevated CO_2_ and Temperature on F. graminearum Disease Severity in Corn

**Figure 4:**
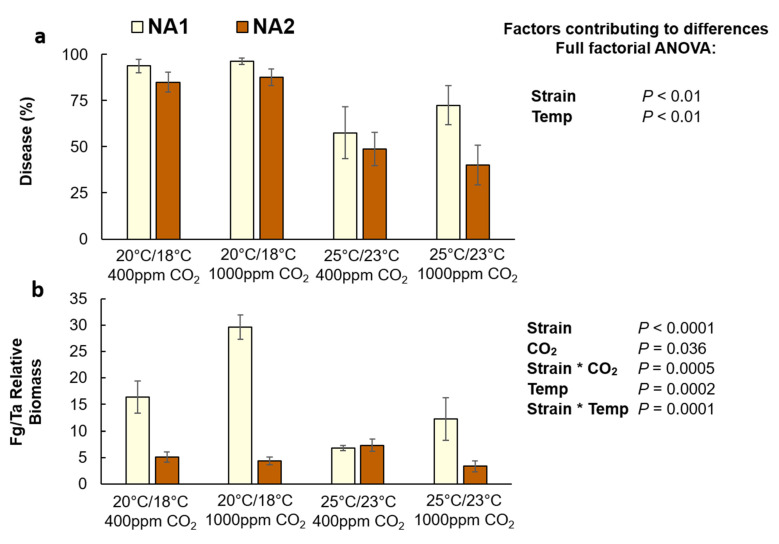
Disease severity (**a**) and relative fungal biomass (Fg/Ta) (**b**) in *Fusarium graminearum* inoculated Gaspe Flint corn grown at ambient and elevated carbon dioxide and temperature. Corn inoculated with the NA1 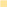 strain 13MN1-6, and the NA2 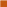 strain 12SD6-2 (*n* = 8). Interaction plots were generated in JMP demonstrating correlations (Supplemental Figure S3).

Strain and temperature significantly contributed to visual disease symptoms in corn according to the full-factorial ANOVA (Figure 4a). Unlike in wheat, disease symptoms in corn were 40% less at the warm temperature treatment in comparison to the cool treatment (Figure 4a; *p* < 0.01). Furthermore, the NA1 strain caused 22% more disease compared to the NA2 strain (*p* < 0.01), which was the inverse of observations in wheat.

Overall, the relative NA1 biomass was also significantly greater than the NA2 biomass (Figure 4b, *p* = 0.0001). *F. graminearum* biomass was also greater at elevated CO_2_ (*p* = 0.036), but the effect was dependent on the strain. While the biomass of the NA2 strain was unaffected by elevated CO_2_, the NA1 fungal biomass nearly doubled at elevated CO_2_ (*p* = 0.0005), regardless of temperature treatment. As with disease symptoms, the amount of *F. graminearum* biomass was 46% less in corn at the warm temperature treatment in comparison to the cool temperature, and the effect of temperature was significantly more severe for the NA1 strain (*p* = 0.0001).

**Figure 5:**
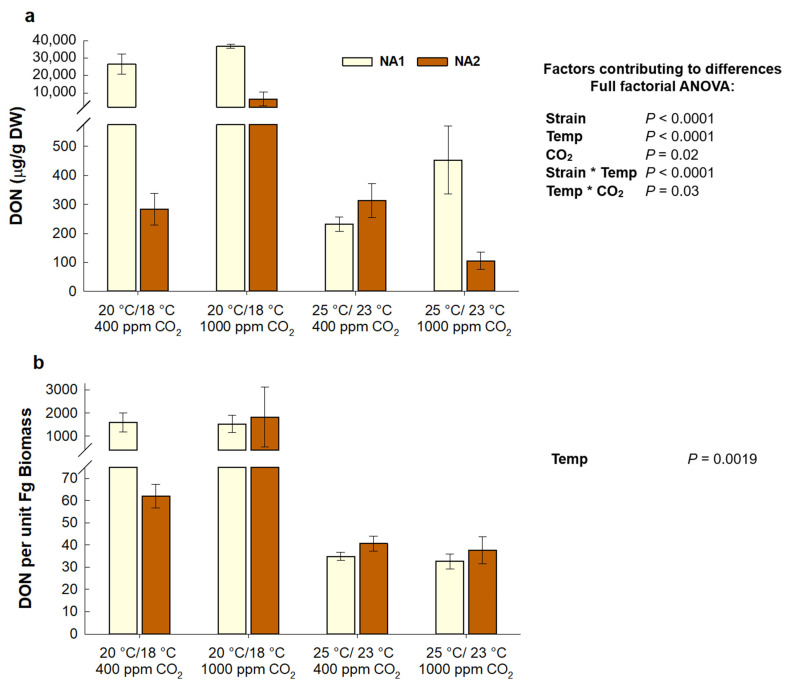
DON accumulation (**a**) and DON per unit fungal biomass (Fg) (**b**) in *Fusarium graminearum* inoculated Gaspe Flint corn grown at ambient and elevated carbon dioxide and temperature. Corn inoculated with the NA1 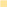 strain 13MN1-6, and the NA2 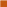 strain 12SD6-2 (*n* = 8). Interaction plots were generated in JMP demonstrating correlations (Supplemental Figure S4).

Factors contributing to a significant difference in corn DON contamination included strain, temperature, CO_2_, and the interaction between strain and temperature and temperature and CO_2_ (Figure 5a). DON contamination was significantly higher in corn inoculated with NA1 (*p* < 0.0001), at the cooler temperature treatment (*p* < 0.0001), and at elevated CO_2_ (*p* = 0.02). The highest amount of DON was in corn at the cool temperature treatment, particularly with the NA1 strain, which produced significantly more DON than the NA2 strain (*p* < 0.0001). The difference in DON contamination of corn between the temperature treatments was the most dramatic, with DON content being 99% and 94% less at the warmer treatment for the NA1 and NA2 strain, respectively. At elevated CO_2_, DON contamination was not significantly impacted at the warm temperature treatment but was approximately 1.4 and 23 times greater in cool temperature treated corn inoculated with the NA1 and NA2 strain, respectively (*p* = 0.03).

Furthermore, DON production per unit *F. graminearum* biomass was significantly less in corn at the warm temperature treatment (Figure 5b; *p* = 0.0019). However, neither *F. graminearum* strain nor CO_2_ concentration affected DON production per unit biomass in corn.

## 3. Discussion

Our findings, that elevated CO_2_ exacerbated *Fusarium* disease outcomes, are consistent with previous reports. Elevated CO_2_ was shown to increase disease susceptibility in wheat, resulting in increased disease damage and DON contamination in a host cultivar and pathogen strain dependent manner [8,16,19,20]. Furthermore, elevated CO_2_ can also significantly alter plant nutritional content, particularly in C3 photosynthetic crops [21,30,31]. Changes in the host nutrient profile may have reduced *F. graminearum* growth but increased DON per unit biomass, as previously observed in *F. graminearum* infected grain from wheat grown at elevated CO_2_ [17]. Our current study found a significant increase of DON contamination in infected wheat that was grown at elevated CO_2_ (Figure 3). Furthermore, the NA2 strain caused more disease damage at elevated CO_2_ in wheat, compared with the NA1 strain. The increased aggressiveness of the NA2 strain to elevated CO_2_ was only observed in inoculated wheat, whereas in corn the NA1 strain had significantly greater fungal biomass in response to elevated CO_2_ (Figure 4). Differences in corn and wheat disease outcomes are not likely due to *F. graminearum* strain host origin, as previous studies have shown that *F. graminearum* strains which were isolated from either wheat or corn had no observable host preference in terms of disease aggressiveness and DON accumulation [13].

Though there was no change in observable disease damage in corn grown at elevated CO_2_, the amount of DON was greater, particularly at the cool temperature with elevated CO_2_. While C4 photosynthesis and plant growth benefit little from elevated atmospheric CO_2_, higher CO_2_ concentrations have been observed to increase the disease severity of *Fusarium verticillioides* in corn due to changes in secondary metabolite responses [32]. Interestingly, *F. verticillioides* increased fungal biomass at elevated CO_2_ without a corresponding increase in mycotoxin accumulation or production. Herein we show that *F. graminearum* similarly accumulated greater amounts of fungal biomass in corn at elevated CO_2,_ but this was also accompanied by increased DON contamination (Figure 4 and Figure 5). While elevated CO_2_ altered DON contamination levels in both wheat and corn, the largest factor in disease outcome and mycotoxin contamination was temperature.

Temperature and humidity are key factors in the likelihood, and severity, of FHB outbreaks and mycotoxin contamination in cereal crops [10]. We consistently found that the warmer temperature treatment suppressed *F. graminearum* biomass and DON contamination in both wheat and corn. However, at the warmer temperature there was greater visual disease symptoms in wheat. Previous research has shown that visual disease is often poorly correlated with fungal biomass and yield loss in wheat [33]. We further observed strain specific differences in response to temperature, as the NA2 strain was more aggressive and caused more disease symptoms compared to NA1 at warm temperatures (Figure 2a). However, despite greater observable disease severity, the NA2 strain produced less toxins at the warm temperature treatment than in the cool treatment (Figure 3a). In vitro, the optimum temperature for *F. graminearum* growth has been reported to be 25 °C, with optimal pathogenicity between 20–25 °C; though *F. graminearum* temperature response was significantly impacted by the geographic origin of the isolate [34]. Interestingly, our current results show that in *planta* the warmer temperature treatment of 25 °C/23 °C was less optimal than the cool treatment 20 °C/18 °C with respect to *F. graminearum* biomass accumulation and DON contamination (Figure 2b or Figure 3b). However, despite the reduced pathogen biomass, the overall DON production per unit *F. graminearum* biomass significantly increased at warmer temperatures in wheat (Figure 3b). This was not the case in corn (Figure 5b), where DON production by *F. graminearum* was suppressed by 97% in warmer growing conditions, contrary to what was observed in vitro, where the optimal production of DON by *F. graminearum* on sterilized corn grain was found to be approximately 25 °C [11].

The incongruity between the in vitro and in *planta* pathogen temperature response suggests a complex host × pathogen interaction. Our results demonstrate that growing temperature has a substantial impact on all aspects of *F. graminearum* infection in both wheat and corn, as it was the only factor which was significant in every comparison.

The reduction of fungal biomass in corn at elevated temperatures may ultimately be quite beneficial in reducing FHB incidence in wheat. Corn/wheat crop rotations typically result in significant FHB disease incidence, as field corn residues greatly increase the fungal inoculum present during the following wheat growing season [35,36]. Warm temperature not only reduced *F. graminearum* biomass in corn (Figure 4a), but could further reduce the rate of infection, as the optimal temperature for perithecia formation is 21.7 °C, and formation decreases with increasing temperatures until complete failure above 30 °C [37]. However, changes in climate and temperature can rapidly shift pathogen populations, in corn, *F. verticillioides* and *Aspergillus flavus* will outcompete *F. graminearum* under drier, warmer, growing conditions [38,39]. Therefore, while the reduction in disease, fungal biomass, and DON accumulation at elevated temperatures is promising news, increased growing temperatures could also promote infection by far more dangerous mycotoxigenic fungal pathogens like *Aspergillus flavus*, which produces carcinogenic aflatoxins [40,41].

## 4. Materials and Methods

### 4.1. Wheat and Corn Cultivars and Growth Conditions

Two short-stature, rapidly developing cultivars of wheat and corn were selected for analysis. Apogee seed was kindly provided by Bruce Bugbee at Utah State University [28]. The Gaspe Flint seed [29] was provided by Mark Busman with the USDA ARS Mycotoxin Prevention and Applied Microbiology Unit in Peoria IL. Both cultivars were propagated in a temperature-controlled greenhouse prior to growth chamber experiments.

To evaluate the effects of elevated CO_2_ and temperature on *F. graminearum* infection and mycotoxin contamination a 2 × 2 × 2 full factorial experiment was designed with the factors of two temperature treatments (20 °C/18 °C and 25 °C/23 °C), two CO_2_ concentrations (400 ppm and 1000 ppm), and two *F. graminearum* strains (13MN1-6 and 12SD6-2). The wheat cultivar Apogee, and the corn cultivar Gaspe Flint, were grown in Conviron PGR15 environmentally controlled growth chambers (Controlled Environments Inc., Winnipeg, MB, Canada). Apogee and Gaspe Flint both grow well under similar control conditions (between 18–25 °C) allowing for simultaneous growth, and the short stature of the Gaspe Flint cultivar was ideal for the limited vertical space within the growth chamber. Eight wheat seeds, or four corn seeds, were sown in a 20 × 15 cm plastic pot, filled with approximately 4 L of SunGrow Horticulture potting mix (Agawam, MA, USA). After one week the plants were culled to 5 plants per pot for wheat, and 2 plants per pot for corn. Growth chamber conditions were set to either ambient CO_2_ (approximately 400 ppm, a[CO_2_]) or elevated CO_2_ (1000 ± 10 ppm, e[CO_2_]), with 50 ± 10% relative humidity and a 14 h photoperiod (550 μmol m^−2^ s^−1^ photosynthetic photon flux density). Chamber temperatures were set to either 20 °C/18 °C, day and night, or 25 °C/23 °C, day and night, respectively. Plants were watered daily, and pot positions were randomized weekly within the growth chamber. Additionally, a biweekly fertilization supplement, using soluble Peters 20-20-20 (The Scotts Company, Marysville, OH, USA) was applied until anthesis, or pollination, for wheat and corn, respectively.

### 4.2. Inoculations and Disease Evaluation

Two *F. graminearum* isolates, 13MN1-6 and 12SD6-2, representing an NA1 and NA2 strain [42], respectively, were used to inoculate wheat and corn (Table 1). NA1 isolates produce 15-acetyl-deoxynivalenol (15-ADON) and NA2 isolates produce 3-acetyl-deoxynivalenol (3-ADON) [43], but both of these metabolites are converted into DON within the plant [13,14]. Media preparation and inoculations were performed according to previously reported methodology [19]. In brief, fungal isolates from glycerol stock were grown on V8 agar plates for 7 d before an agar plug was transferred into 20 mL of mung bean broth to promote conidia formation. Cultures were grown for 48 h, at 28 °C, under dark conditions in a New Brunswick Innova 44 incubator shaker (Eppendorf, Hauppauge, NY, USA). The cultures were briefly centrifuged, and the supernatant discarded. Afterwards, a 1 × 10^5^ mL^−1^ microconidia suspension was produced by the addition of 0.04% Tween 20 in sterile water (Thermo Fisher Scientific, Waltham, MA, USA), and subsequently used for inoculations.

Apogee was inoculated at flowering, anthesis, with 10 µL of the conidial suspension into single florets (biological replicates: *n* = 12) following previously reported methodology [44]. Immediately afterwards, a plastic bag was placed onto the inoculated wheat heads to maintain a high humidity environment for 3 d. Disease progression and the AUDPC in Apogee was determined by visually assessing the number of diseased florets, bleached or necrotic plant tissue, 7, 10, 14, and 17 days after inoculation [45]. Disease severity in wheat was determined by the ratio of diseased florets to the total florets on the inoculated wheat head. At day 17, the infected wheat heads were collected and stored at −80 °C for further analysis.

Gaspe Flint corn was inoculated 5 d after manual pollination with 1 mL of the conidial suspension into each cob (biological replicates: *n* = 8) injecting the inoculum into the side of the ear following previously reported methodology [29,32]. Disease severity was visually scored in Gaspe Flint 17 days post inoculation following previously reported protocols [46]. Afterwards, cobs were collected and stored at −80 °C for further analysis. All inoculations and disease evaluations were experimentally replicated.

### 4.3. Mycotoxin Analyses

Mycotoxins, were extracted from 1 g of ground infected plant tissues, derivatized, and analyzed via GC-MS, on an Agilent 7890 gas chromatograph (Agilent Technologies, Wilmington, DE) fitted with a HP-5MS column (30 m, 0.25 mm, 0.25 μm) and a 5977 mass detector following previously reported methodology [47]. Though the two *F. graminearum* strains produced two distinct acetylated forms of deoxynivalenol (3-ADON and 15-ADON) in liquid media, in *planta* the mycotoxin deoxynivalenol (DON) was the overwhelmingly predominant form [26]. Therefore, only the accumulation of the mycotoxin DON in infected plant tissues was evaluated for this manuscript.

### 4.4. Estimation of Host and Pathogen Biomass

The relative amount of fungal biomass in the inoculated tissues was assessed using the ratio of *F. graminearum* DNA to plant host DNA via a quantitative polymerase chain reaction (qPCR), following the previously reported protocols [32,45]. Four technical replications were performed per assay; primers and probes can be found in Table 2. The relative amount of *F. graminearum* DNA to host DNA was determined by dividing the geometric mean of initial DNA concentration (N_0_) from the *Fusarium* probes by the geometric mean of N_0_ from the host probes. The amount of DON relative to *F. graminearum* DNA was estimated by dividing the µg g^−1^ DON by the relative pathogen biomass, as quantified by qPCR.

### 4.5. Statistical Analyses

Results were evaluated by a 2 × 2 × 2 full factorial analysis of variance (α = 0.05; JMP V15.0), to determine the significant differences in the disease and mycotoxin contamination of hosts due to the effects of temperature and elevated CO_2_. Data from the wheat and corn hosts were analyzed separately. Details of factor combinations and significant interactions can be found within the figures.

## 5. Conclusions

Elevated CO_2_ was determined to significantly increase the accumulation of the mycotoxin deoxynivalenol in infected plants. Furthermore, infected plants in cool growing conditions had the highest amounts of pathogen biomass and toxin accumulation in both wheat and corn. Warm temperatures suppressed pathogen growth and toxin accumulation, with reductions as great as 99% in corn. In wheat, despite reduced pathogen biomass and toxin accumulation at warm temperatures, *F. graminearum* was more aggressive with greater disease damage and toxin production per unit biomass. However, we found that atmospheric CO_2_ and temperature had essentially no significant interactions, except for greatly increased deoxynivalenol accumulation in corn at cool temperatures and elevated CO_2_. This work helps elucidate the complex interaction between abiotic stresses and biotic susceptibility to *Fusarium graminearum* infection in wheat and corn to better understand the potential impact global climate change poses to future food security.

## Figures and Tables

**Table 1:** *Fusarium graminearum* strains used for inoculations and disease assays. Strains are distinguished from one another by North American population group [25] and mycotoxin chemotype [26].

*F. graminearum* Strains	13MN1-6	12SD6-2
North American *F. graminearum* population [25]	NA1	NA2
Mycotoxin Chemotype [26]	15-acetyl-deoxynivalenol (15-ADON)	3-acetyl-deoxynivalenol (3-ADON)
	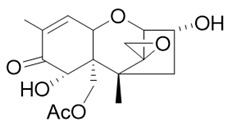	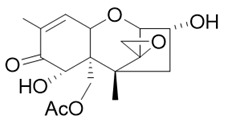

**Table 2:** Primer and probe sequences used for qPCR amplification. Three sets of primers and corresponding probes were used for quantification of relative *F. graminearum* to wheat, or corn, biomass. Asterisks (*) indicate primer sequences specifically developed for this study.

Primer Name	Organism	Gene Product	Primer Sequence	Reference
Zm.GAPDH Forward	*Z. mays*	Glyceraldehyde-3-phosphate dehydrogenase	CGAGAATAAATGTGGATGGCG	*
Zm.GAPDH Reverse	*Z. mays*	Glyceraldehyde-3-phosphate dehydrogenase	GCAGGAAGGGAAACAAAAGTG	*
Zm.TUB Forward	*Z. mays*	Tubulin	TCCACATTCATCGGCAACTC	*
Zm.TUB Reverse	*Z. mays*	Tubulin	AACTCCATCTCATCCATGCC	*
Zm.CYP Forward	*Z. mays*	Peptidyl-prolyl cis-trans isomerase	CGTCCGTTCCTTTGGATCTG	*
Zm.CYP Reverse	*Z. mays*	Peptidyl-prolyl cis-trans isomerase	GAAACACGAATCAAGCAGAGG	*
Fg.Tri101 Forward	*F. graminearum*	Trichothecene 3-*O*-acetyltransferase	GGACTCTGGGATTACGACTTTG	[17]
Fg.Tri101 Reverse	*F. graminearum*	Trichothecene 3-*O*-acetyltransferase	ATCAGGCTTCTTGGGCATAAA	[17]
Fg.TEF Forward	*F. graminearum*	Translation elongation factor	CAGTCACTAACCACCTGTCAAT	[17]
Fg.TEF Reverse	*F. graminearum*	Translation elongation factor	AATGGTGATACCACGCTCAC	[17]
Fg.RED Forward	*F. graminearum*	Reductase	TGACAGCTTTGGTTGTGTTTG	[17]
Fg.RED Reverse	*F. graminearum*	Reductase	CTTGGCTGGAATGAGTCTGT	[17]
Ta.Ef1 Forward	*T. aestivum*	Elongation factor	GATTGACAGGCGATCTGGTAAG	[17]
Ta.Ef1 Reverse	*T. aestivum*	Elongation factor	GGCTTGGTGGGAATCATCTT	[17]
Ta.Actin Forward	*T. aestivum*	Actin	CCAAGGCCAACAGAGAGAAA	[17]
Ta.Actin Reverse	*T. aestivum*	Actin	GCTGGCATACAAGGACAGAA	[17]
Ta.PAL Forward	*T. aestivum*	Phenylalanine ammonia-lyase	GTGTTCTGCGAGGTGATGAA	[17]
Ta.PAL Reverse	*T. aestivum*	Phenylalanine ammonia-lyase	GTATGAGCTTCCCTCCAAGATG	[17]

## Data Availability

Data presented in this study is available from the corresponding author on reasonable request.

## Supplementary Materials

The following are available online at https://www.mdpi.com/article/10.3390/plants10122582/s1, Figure S1: Interaction plots generated in JMP demonstrating correlations of variables, Figure S2: Interaction plots generated in JMP demonstrating correlations of variables, Figure S3: Interaction plots generated in JMP demonstrating correlations of variables, Figure S4: Interaction plots generated in JMP demonstrating correlations of variables.
