# Supplementary material for: Effects of Atmospheric CO2 and Temperature on Wheat and Corn Susceptibility to Fusarium graminearum and Deoxynivalenol Contamination
Source: Plants (Basel). 2021 Nov 25;10(12):2582. doi: 10.3390/plants10122582 (PMC8709488; doi:10.3390/plants10122582)
Supplement: Supplementary file 1 [file plants-10-02582-s001.zip › plants-1476126-supplementary.pdf]

Supplemental Information:

TITLE: Effects of atmospheric CO_2 and temperature on wheat and corn susceptibility to *Fusarium graminearum* and deoxynivalenol contamination.

William T. Hay[1*], Susan P. McCormick[1], Martha M. Vaughan[1]

[1] USDA, Agricultural Research Service, National Center for Agricultural Utilization Research, Mycotoxin Prevention and Applied Microbiology Research Unit, 1815 N. University, Peoria, IL 61604, USA

*Corresponding author: William.Hay@usda.gov ; ORCID ID: 0000-0001-8784-6591

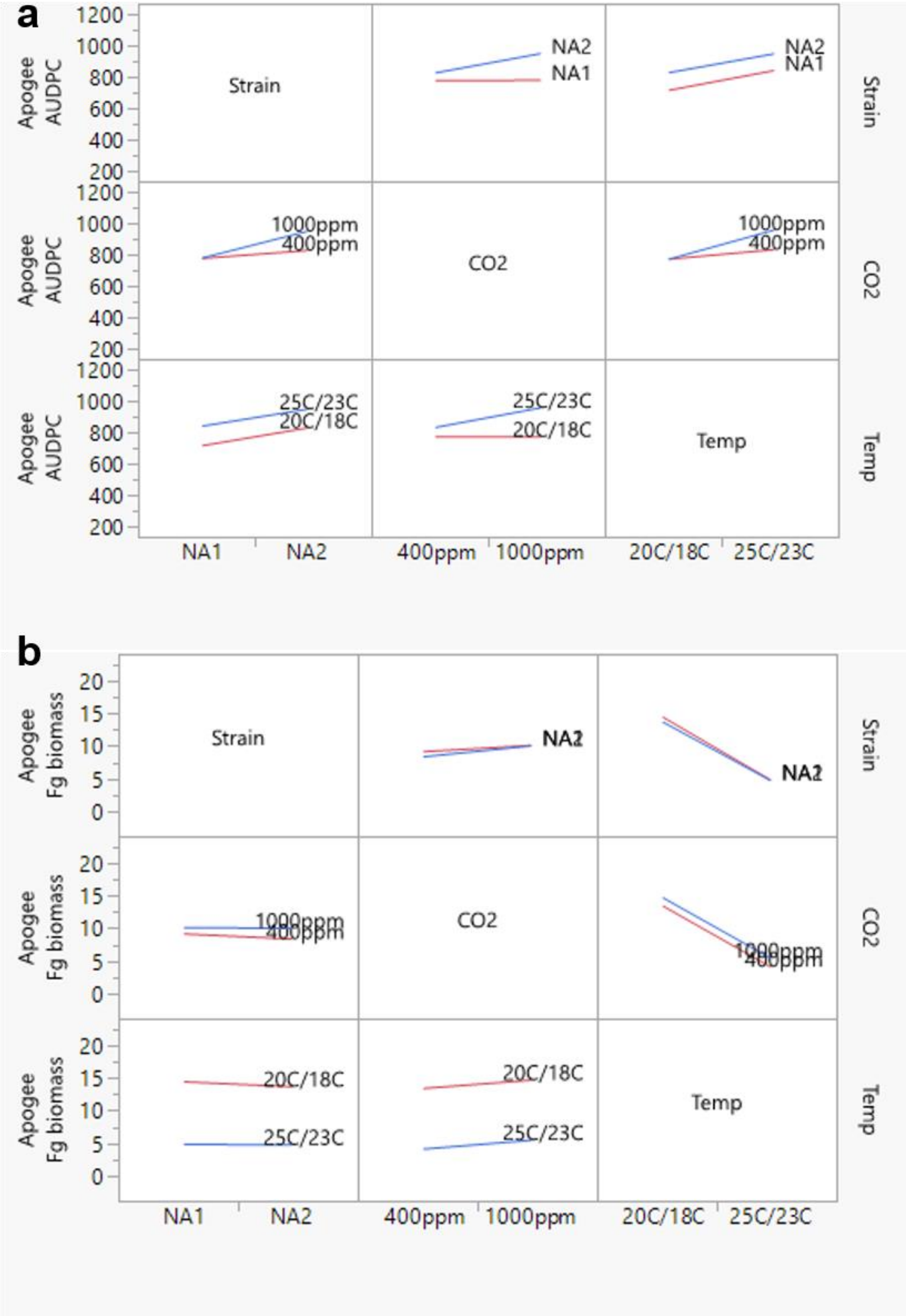

Supplemental Figure S1. Interaction plots generated in JMP demonstrating correlations of variables presented in Figure #2 in the main manuscript.

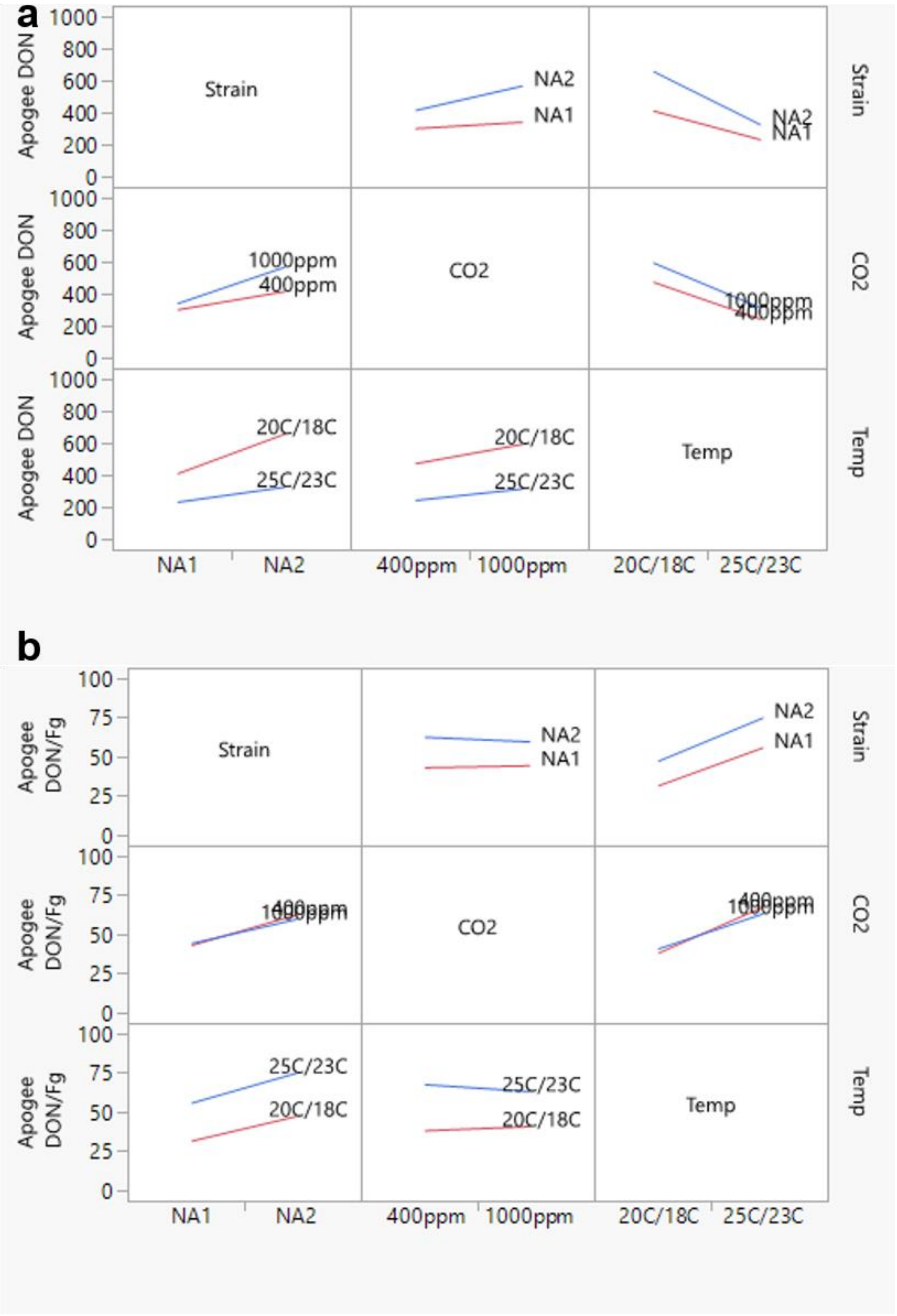

Supplemental Figure S2. Interaction plots generated in JMP demonstrating correlations of variables presented in Figure #3 in the main manuscript.

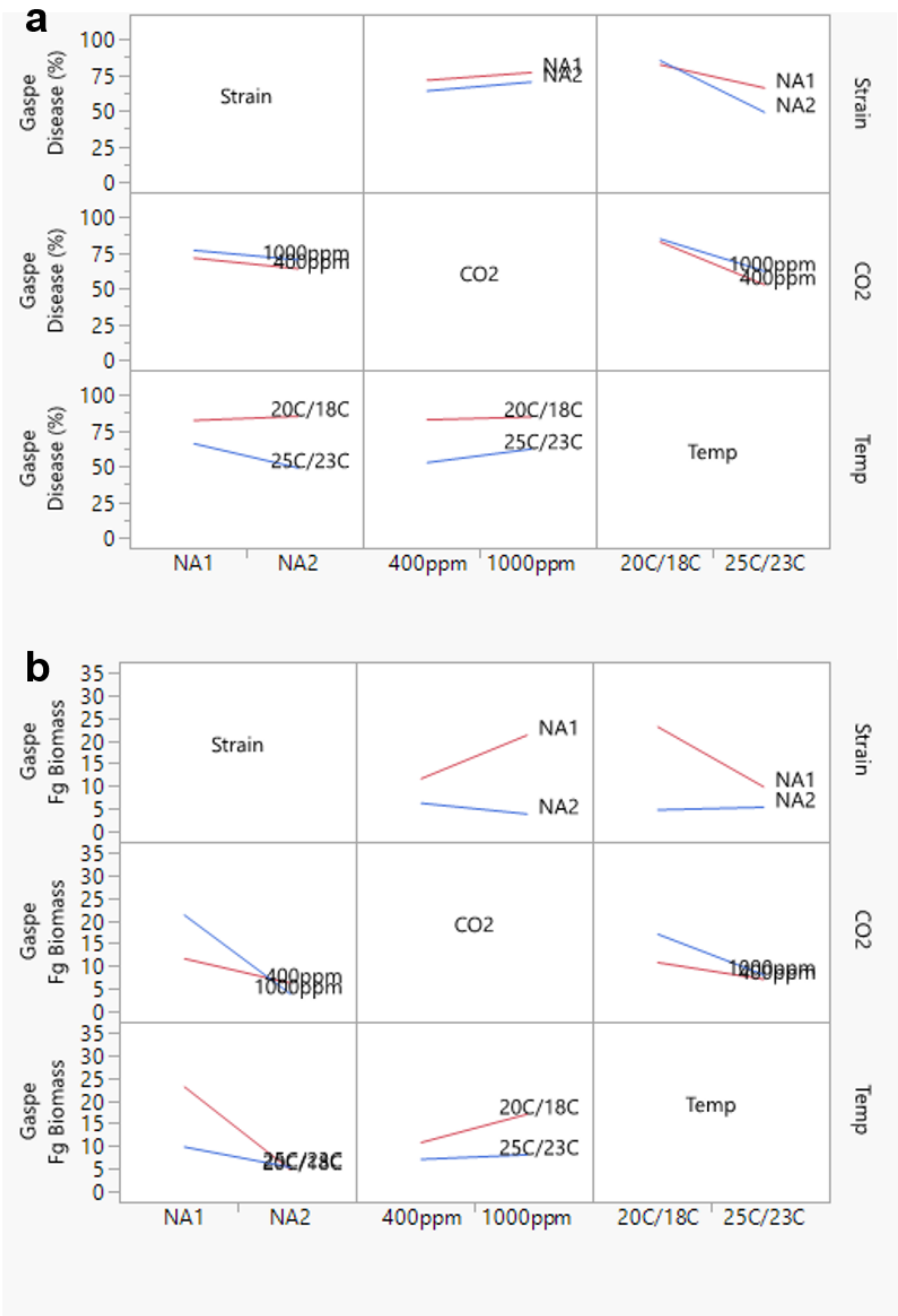

Supplemental Figure S3. Interaction plots generated in JMP demonstrating correlations of variables presented in Figure #4 in the main manuscript.

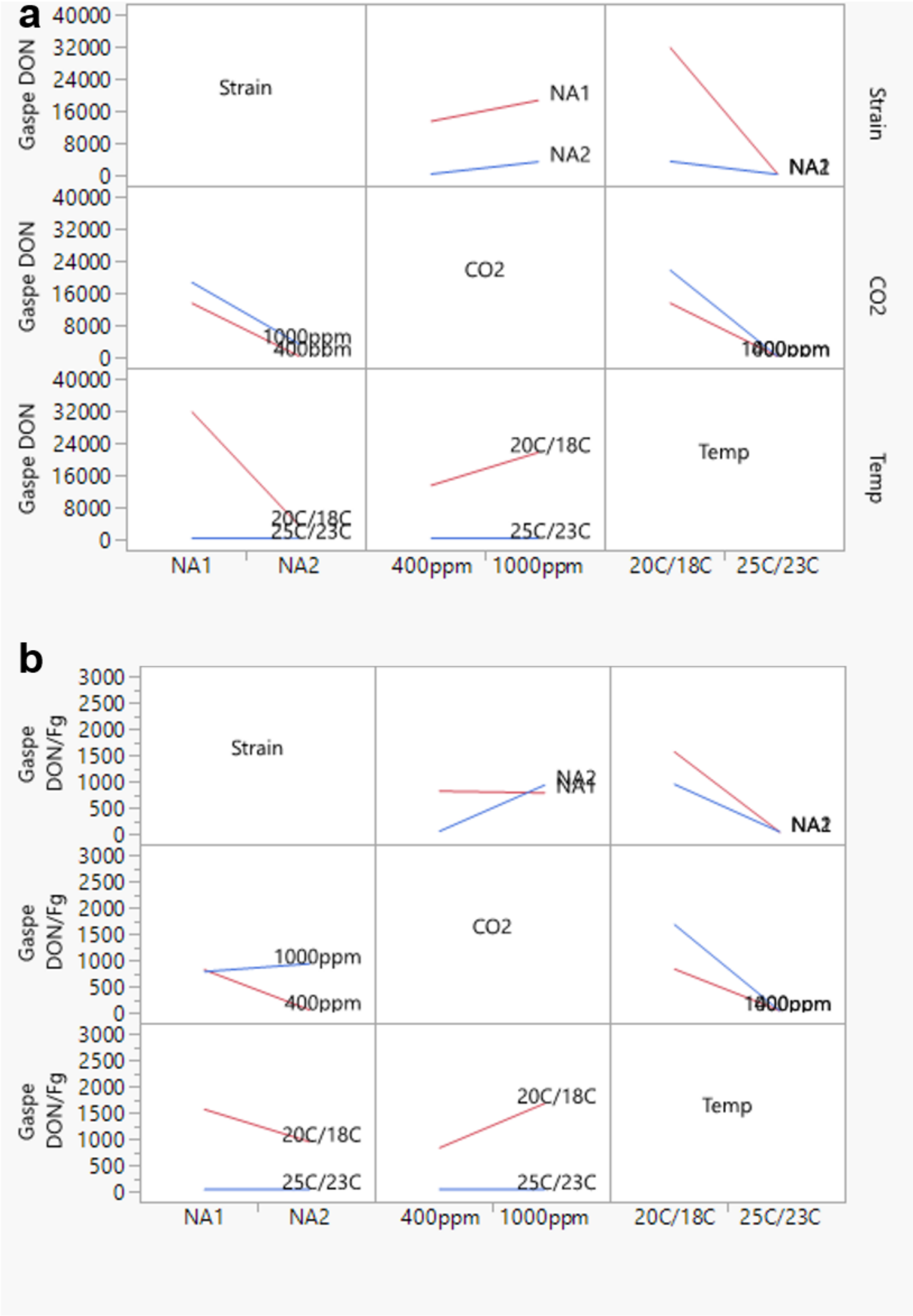

Supplemental Figure S4. Interaction plots generated in JMP demonstrating correlations of variables presented in Figure #5 in the main manuscript.
